# Antifungal Drug Susceptibility Profile of *Candida kefyr* Isolated from Clinical Samples and Dairy Products

**DOI:** 10.1155/2024/6594366

**Published:** 2024-09-23

**Authors:** Zahra Zareshahrabadi, Samin Khaliji, Maryam Roudbari, Kamiar Zomorodian

**Affiliations:** ^1^ Basic Sciences in Infectious Diseases Research Center Shiraz University of Medical Sciences, Shiraz, Iran; ^2^ Department of Medical Parasitology and Mycology Shiraz University of Medical Sciences, Shiraz, Iran; ^3^ Department of Parasitology and Mycology School of Medicine Iran University of Medical Sciences, Tehran, Iran

## Abstract

Exploring drug susceptibility is a critical endeavor in the scientific community, setting the stage for advancements in understanding and combating various pathogens. *Candida kefyr* has emerged as a significant pathogen, particularly affecting immunocompromised individuals with hematologic malignancies and HIV/AIDS conditions. This study aimed to assess the antifungal susceptibility profile of *Candida kefyr* isolates obtained from clinical samples and dairy products. A total of 134 *Candida kefyr* yeast isolates were retrieved from three distinct groups: (1) healthy individuals (*n* = 41), (2) patients (*n* = 24) including hematologic malignancy (*n* = 9), HIV/AIDS (*n* = 7), and diabetes (*n* = 8), (3) dairy products (milk, yogurt, and cheese, *n* = 69) stored at −70°C in the Shiraz University of Medical Science. All *Candida kefyr* isolates were previously identified using conventional and molecular methods. Susceptibility to antifungal drugs, including caspofungin, fluconazole, itraconazole, voriconazole, and amphotericin B, was determined using the microdilution method following CLSI-M27-A3 protocols, with results interpreted according to CLSI-M27-S4 guidelines. The study emphasizes the clear variation in antifungal susceptibility testing of *Candida kefyr* strains when compared across different groups, including patients, healthy people, and dairy products. According to the results, across all groups, a high minimum inhibitory concentration of fluconazole is evident, and healthy individuals show the highest minimum inhibitory concentration geometric means (4.0681). Also, 79.1% of the isolates were wild type to amphotericin B, with the lowest minimum inhibitory concentration compared to other antifungals tested. This suggests that amphotericin B was more effective against *Candida kefyr*. These findings showed fewer susceptibilities of *Candida kefyr* to both triazole and echinocandin classes of antifungal agents. Additionally, it is noteworthy that individuals without medical conditions exhibited higher minimum inhibitory concentration rates to these antifungal agents in comparison to those with underlying health conditions. Consequently, timely diagnosis and appropriate therapeutic interventions emerge as imperative in the effective management of candidiasis cases.

## 1. Introduction

Over the past two decades, there has been a significant increase in the prevalence and severity of fungal infections. This rise is particularly noticeable among immunocompromised individuals, such as those with weakened immune systems due to conditions like HIV/AIDS, cancer, or organ transplants, and patients hospitalized with severe underlying illnesses. These infections pose a major challenge due to their often invasive nature, leading to complications in clinical management and treatment. Among fungal pathogens, species belonging to the *Candida* genus are most frequently identified as causative agents [1–4]. Despite five predominant species *Candida albicans, Candida glabrata, Candida parapsilosis, Candida tropicalis*, and *Candida krusei* accounting for over 90% of *Candida* infections, the continually expanding list of reported species underscores the evolving landscape of fungal pathogens [5, 6]. Laboratories increasingly strive to identify *Candida* strains at the species level, a practice essential for optimizing treatment strategies against *Candida* infections [7–10]. *Candida kefyr* (*C. kefyr*), formerly recognized as *Candida pseudotropicalis,* represents a yeast with its teleomorph identified as *Kluyveromyces marxianus*. The ecological niche of this yeast remains incompletely elucidated, although evidence suggests its adaptability to diverse environments, including dairy products. *C. kefyr* is interesting from a scientific and medical perspective due to its presence in various ecological niches and its potential role in causing infections in humans [11, 12]. Recently, *C. kefyr* has garnered attention as a potential emerging pathogen linked to bloodstream infections, and resistance to antifungal agents like fluconazole and amphotericin B has been observed in some *C. kefyr* isolates. Given the relatively infrequent nature of this yeast, which may evolve into a clinically significant pathogen in the future, a comprehensive understanding of its in vitro susceptibility to both established and novel antifungal agents is crucial for informing potential treatment modalities [8, 12, 13]. The growing resistance to antifungal medications, along with the emergence of new, more resilient fungal strains, further exacerbates this issue. The Clinical and Laboratory Standards Institute (CLSI) developed new *Candida* species–specific clinical breakpoints for some antifungal agents, like fluconazole, voriconazole, and echinocandins [14–16]. It is important to acknowledge that established clinical breakpoints are not available for *C. kefyr* and antifungal drugs. Therefore, categorizing the isolates as susceptible or resistant is not possible. However, the minimum inhibitory concentration data were interpreted using the reported epidemiological cutoff value classified the isolate as Non–wild type and Wild type. This trend underscores the need for enhanced surveillance, improved diagnostic techniques, and the development of new antifungal therapies to effectively combat these increasingly common and complex infections [16]. The primary objective of this study was to investigate the antifungal susceptibility profile of five distinct antifungal agents against a collection of clinical and dairy product isolates of *C. kefyr*. By studying the antifungal susceptibility profiles of *C. kefyr* isolates from dairy products, we can gain insights into the potential role of these products in the transmission of *C. kefyr* infections. Additionally, comparing susceptibility profiles of isolates from clinical and dairy sources can reveal potential differences and inform strategies for managing infections. Furthermore, understanding the antifungal susceptibility of *C. kefyr* isolates from dairy products can contribute to public health surveillance efforts to monitor the emergence of antifungal resistance in this species. By doing so, the current study aims to contribute valuable insights into the management of infections caused by this emerging pathogen, providing a foundation for informed therapeutic decision-making in clinical settings.

## 2. Methods

### 2.1. Study Design, Clinical and Dairy *C*. *kefyr* Isolates

A total of 134 *C. kefyr* isolates were obtained from three groups, including (1) healthy individuals (*n*: 41), (2) patient's individuals (*n*: 24), and (3) dairy products (*n*: 69), which were available in the −70°C freezer at the Shiraz University of Medical Sciences and had been previously identified to the genus and species levels using CHROMagar *Candida* and rDNA PCR amplification. The details of the participants in the current study are as follows: Healthy individuals, comprising 28 oral and 13 vaginal samples. Patients, including hematologic malignancy (*n*: 9), diabetes (*n*: 8), and HIV/AIDS (*n*: 7). Dairy product samples, consisted of 49 samples of yogurt, 19 samples of milk, and one sample of cheese. All clinical and dairy samples were cultured on Sabouraud dextrose agar (SDA, Merck, Germany) supplemented with chloramphenicol (50 mg/L) plates and incubated for 48 h at 32°C to check the purity of the colonies. A reference strain of *C. kefyr* (ATCC 38296) was used for quality control.

### 2.2. Clinical Characteristics

In this study, a total of 24 clinical samples from patients were examined, of which 14 (58%) were obtained from males and 10 samples (42%) were obtained from females and included in the study. The mean age of the studied samples with *C. kefyr* ranged from 17 to 73 years. The individuals participating in this study were evaluated for underlying medical conditions, among which 7 individuals (29%) had HIV/AIDS, 9 individuals (37.5%) had hematologic malignancy, and 8 individuals (8.5%) had diabetes. The HIV/AIDS patients consisted of 6 males and 1 female, with an average age ranging from 36 to 45 years, who were diagnosed with oral candidiasis with *C. kefyr*. Lymphocyte (T-cell) counts for these patients ranged from 120 to 730 cells per cubic millimeter. Among this group, 4 patients (57%) had a history of oral candidiasis with symptoms including erythematous patches, pseudomembranous plaques, and angular cheilitis, and 3 individuals (43%) had no history of previous oral candidiasis. The most common clinical symptom observed in this group was pseudomembranous plaques (43%). They were treated with medications such as nystatin, fluconazole, and itraconazole, as documented in their medical records, and those received fluconazole combination with itraconazole (15%) were treated. The hematologic malignancy patients included 5 males (56%) and 4 females (44%), with an average age ranging from 28 to 73 years, all of whom had oral candidiasis. Among them, 5 cases (56%) were diagnosed with acute myeloid leukemia (AML), 2 cases (22%) had chronic myeloid leukemia (CML), and 2 cases (22%) had chronic lymphocytic leukemia (CLL). All of them were undergoing chemotherapy with arsenic trioxide, cytarabine, fludarabine, and rituximab. Within this group of patients, 7 individuals (78%) had a history of previous oral candidiasis, while 2 individuals (22%) had no history of oral candidiasis in their medical records. Clinical symptoms in this group included erythematous patches, pseudomembranous plaques, angular cheilitis, and ulceration. The most common clinical symptom observed in this group was erythematous patches (66.6%). The diabetic patients comprise a total of 8 cases, including 5 females (62%) and 3 males (38%), with ages ranging from 17 to 63 years. These clinical samples were obtained from various sources, with 4 cases (50%) from the oral cavity, 3 cases (38%) from urine, and 1 case (12%) from nail. The healthy individuals group consists of 41 samples, which were obtained from the oral cavities of healthy males and females with an age range of 21 to 65 years.

### 2.3. Characteristics of Dairy Samples

The *C. kefyr* samples were isolated from a total of 69 dairy products, which included 49 samples (71%) from yogurt, 19 samples (27%) from milk, and 1 sample (2%) from cheese.

### 2.4. Antifungal Susceptibility Testing

Susceptibility testing followed the recommendations proposed by the Clinical and Laboratory Standards Institute (CLSI-M27-A3) for yeasts with results interpreted according to CLSI-M27-S4 guidelines. Powders of amphotericin B (AMB, Sigma-Aldrich USA), voriconazole (VOR, Sigma-Aldrich USA), itraconazole (ITR, Sigma-Aldrich USA), caspofungin (CAS, Sigma-Aldrich USA), and fluconazole (FLU, Sigma-Aldrich USA) were obtained from the respective manufacturers. Roswell Park Memorial Institute1640 (RPMI-1640) (Sigma, St.Louis, Missouri) was made according to the manufacturer's protocol and buffered to pH 7.0 with 0.165 N-morpholino propanesulfonic acid (MOPS) buffer (Sigma, USA). Stock solutions with a 10-fold concentration for each antifungal were prepared in dimethyl sulfoxide (DMSO) except FLU which is soluble in water. The final concentrations of the working solutions were obtained by using RPMI medium. The final concentrations of the antifungal agents were 0.032 to 16 *μ*g/mL for AMB, VOR, ITR, and CAS; and 0.12–64 *μ*g/mL for FLU [17]. Briefly, testing was performed in flat-bottom microdilution plates with RPMI-1640 medium supplemented with 2% glucose. The inoculum suspensions (0.5 McFarland) were prepared by the spectrophotometric method (at 530 nm) (Pharmacia Biotech Cambridge, England, Ultrospec 3000 UV/visible spectrophotometer), and diluted to 0.5 × 10^3^ to 2.5 × 10^3^ cells/ml using RPMI-1640 medium. A 100 *μ*l volume of *C. kefyr* inoculum and an equal volume of antifungal agents were added to each well. Antifungal drug-free and yeast-free wells were included as positive and negative controls [18]. The MIC values of azoles and echinocandins were determined following the CLSI guidelines using the method of prominent and complete inhibition (80% inhibition) of growth compared to that of the drug-free growth control well. The MIC of AMB was determined using the method of no visible growth compared to that of the drug-free growth control well. For the antifungal tested, the ranges of MIC, MIC50, MIC90, and geometric means (GM), were read and calculated for five antifungal drugs against *C. Kefyr* isolates. This study employed the CLSI broth microdilution method to investigate the antifungal susceptibility profiles of *C. kefyr* isolates. It is important to acknowledge that established clinical breakpoints are not available for *C. kefyr* and antifungal drugs. Therefore, categorizing the isolates as susceptible or resistant is not possible. However, the MIC data were interpreted using the reported epidemiological cutoff value (ECV) classified the isolate as Non–wild type (Non-WT) and Wild type (WT) [19, 20]. A Wild type organism is defined as a strain that does not harbor any acquired resistance to the particular antimicrobial agent being examined for which the MIC results are lower than the ECV. Organisms with acquired or mutational resistance mechanisms may be included among those for which the MIC results are higher than the ECV as a Non-WT (1, 7, 18).

### 2.5. Statistical Analysis

The data collected were expressed as mean and standard deviation for numeric variables, and absolute and relative frequencies for categorical variables. We used the Chi-squared (*χ*^2^) test to analyze categorical variables, and analysis of variance (ANOVA) followed by the Tukey's post-test was used for numeric variables when *p* < 0.05. A significance level of *p* < 0.05 was adopted.

## 3. Result

### 3.1. Antifungal Susceptibility Profile

This study employed the CLSI broth microdilution method to investigate the antifungal susceptibility profiles of *C. kefyr* isolates, and the MIC values were interpreted using the reported ECV. The antifungal susceptibility profile of *C. kefyr* isolates obtained from clinical and dairy samples was assessed using various concentrations of antifungal agents. The MIC values were determined for each drug against each isolate, and the results are presented. The MIC distributions, MIC ranges, MIC50, MIC90, and geometric mean (GM) values of 134 *C. kefyr* isolates tested to 5 antifungal agents are calculated and provided for each group in Tables 1, 2, 3.

The values of MIC50, MIC90, and GM for all isolates used in this study are as follows: Based on previous studies and the CLSI standards, the ECVs for the tested antifungal drugs are defined in Table 4. Following this standard, the results obtained from the drug susceptibility testing can be interpreted as follows: According to the results, among the 134 studied samples, high MIC to FLU is evident across all groups, with healthy individuals showing the highest MIC GM (4.0681), indicating a lower overall FLU susceptibility. Based on the findings, in the patient group, 4 cases, in the healthy individuals group, 19 cases, and in the dairy products, 9 cases showed MIC ≥8 to FLU. Following Tables 1 and 2, among the studied patient group, 2 cases, in the healthy individuals group, 13 cases, and dairy products, 7 cases showed MIC ≥4 to ITR.

In the patients and healthy individuals, 4 cases for each group demonstrated MIC ≥2 to AMB. This study indicated that 31.3% of the isolates were WT to VOR with an MIC range of 0.03–≥16 *μ*g/mL. On the other hand, in this experiment, 79.1% of the isolates were WT to AMB, with the lowest MIC GM and MIC ranges, compared to those of other tested antifungal. These results suggest that AMB, a polyene antifungal drug, is effective against *C. kefyr* isolates.

## 4. Discussion

*Candida* species are responsible for a substantial proportion of healthcare-associated fungal infections on a global scale. In addressing the need to mitigate and manage these infections, it is imperative to promptly identify the most appropriate antifungal treatment options and gain insights into the resistance profiles exhibited by the causative agents against various antifungal agents [21–23]. The emergence of *C. kefyr* as a clinically relevant pathogen has introduced a new layer of complexity to the landscape of healthcare-associated fungal infections. Historically considered an infrequent etiological agent, *C. kefyr* has recently gained prominence in clinical settings, particularly in cases of fungemia [8, 21, 24]. The increased frequency of its isolation in blood cultures, surpassing that of conventionally recognized pathogens, indicates an important shift in its clinical significance [3, 25, 26]. Notably, disseminated infections attributed to *C. kefyr*, also known as *Candida pseudo-tropicalis*, were observed among cancer patients by Reuter et al. [27]. The conceivable relationship between dietary practices, particularly the consumption of dairy products, and the prevalence of severe *C. kefyr* infections presents an intriguing area for inquiry. It has been hypothesized that the presence of these strains in serious human infections might be correlated with dietary habits, as *C. kefyr* has frequently been recovered from various dairy products [28]. *C. kefyr* samples have been recovered from various anatomical sites within the human body, serving as the etiological agent of systemic candidiasis. In previous studies, *C. kefyr* species were isolated from urine and blood samples obtained from patients admitted to the hospital intensive care unit [25, 29]. During a comprehensive epidemiological study conducted in 2020 and based on 2011–2018 year studies, the prevalence of *C*. *kefyr* among clinical yeast isolates was recorded as 0.83% [21]. Furthermore, in the investigation conducted at a medical institution in France, it was observed that among neutropenic patients with acute leukemia, *C. kefyr* was isolated in 17% of cases. These findings highlight the clinical importance and relevance of this *Candida* species [12, 30]. This trend underscores the need for enhanced surveillance, improved diagnostic techniques, and the development of new antifungal therapies to effectively combat these increasingly common and complex infections. The current research aimed to investigate the antifungal susceptibility profile of *C. kefyr* isolates from diverse sources, thereby shedding light on the evolving patterns of resistance. A summary of the activity of five antifungal agents, CAS, FLU, VOR, ITR, and AMB, against *C. kefyr* isolates is presented in Tables 1, 2, and 3. These distributions indicate that *C. kefyr* exhibits lower susceptibility to both triazole and echinocandin antifungal classes, while polyenes, like AMB, remain effective against these isolates. In the present study, among healthy individuals, a higher MIC range to FLU (0.125–≥64 *μ*g/mL) compared to AMB (0.25–2 *μ*g/mL) was observed. Notably, *C. kefyr* strains isolated from healthy individuals displayed a greater degree of drug resistance when compared to those isolated from individuals with underlying diseases. ITR, a triazole antifungal medication, showed a broad range of MIC across the isolated *C. kefyr* samples, ranging from 0.125 to at least 16 *μ*g/mL. Notably, 14.1% (19 out of 134 isolates) exhibited a high MIC (≥16 *μ*g/mL) for ITR. This reduced susceptibility to ITR was found in both clinical samples (including isolates from healthy individuals and diabetic patients) and dairy samples (specifically yogurt). This discrepancy in resistance patterns may be attributed to the immunodeficiency status of the patients and the limited exposure of *C. kefyr* strains to the immune system of these individuals. Our findings also revealed that *C. kefyr* strains isolated from dairy samples exhibited higher drug resistance compared to those obtained from patients with compromised immune systems. This study's finding of higher MIC values, indicating potentially reduced susceptibility, in *C. kefyr* isolates from dairy products compared to clinical isolates warrants further investigation for risk assessment. If confirmed, this data could be crucial for informing stricter food safety regulations concerning the handling and processing of dairy products, particularly those containing unpasteurized or minimally processed milk. Furthermore, public awareness campaigns could be tailored to emphasize proper hygiene practices during food preparation and handling to minimize the risk of *C. kefyr* infections associated with dairy consumption. In a study conducted by Hatice Turk Dagi et al. in Turkey in 2016, *C. kefyr* was isolated from 5% of blood culture samples obtained from patients. Notably, all *C. kefyr* strains demonstrated resistance to AMB [31]. Our study found that AMB was the most effective antifungal drug tested against *C. kefyr* isolates. This is because we observed the lowest percentage (20.9%) of *C. kefyr* isolates that were non-WT for AMB. Isolates that are WT are generally considered susceptible to the antifungal drug. This discrepancy in resistance rates between the two studies underscores potential variations in the prevalence of antifungal resistance among *C. kefyr* strains in different geographical regions or healthcare settings. Our research included seven HIV/AIDS patients diagnosed with oral candidiasis caused by *C. kefyr*. These patients' fungal isolates showed high resistance (non-WT) to FLU, CAS, ITR, and VOR antifungal medications. However, a significant finding was that 85.7% of the *C. kefyr* isolates from these HIV/AIDS patients remained susceptible (WT) to AMB. This suggests that AMB may be a more effective treatment option for *C. kefyr* infections in immunocompromised individuals like those with HIV/AIDS. In a study conducted by Khedri et al. in 2018 [32], which investigated drug susceptibility among various *Candida* species in HIV/AIDS, seven cases of *C. kefyr* were isolated and all of them displayed susceptibility to CAS, FLU, VOR, and AMB. Our study found a higher resistance rate to ITR compared to Khedri et al. study (19.3%) and identified a higher rate of non-WT isolates for ITR, at 62.7%. This suggests that *C. kefyr* isolates in our study population may be less susceptible to ITR. This disparity in drug susceptibility profiles between the two studies suggests potential variations in *C. kefyr* strains or drug resistance patterns within the HIV/AIDS population. The results highlight the importance of considering antifungal susceptibility patterns when selecting treatment options for *C. kefyr* infections. Variability in susceptibility among different clinical groups emphasizes the need for personalized treatment approaches. Monitoring changes in susceptibility patterns over time is crucial for optimizing antifungal therapy. Furthermore, investigations of resistance mechanisms prevalent among *C. kefyr* isolates obtained from both clinical samples and dairy products will be needed.

## 5. Conclusion

This study highlights the need to recognize *Candida kefyr* as a significant but often overlooked pathogen in candidiasis. It reveals key insights into its resistance against commonly used antifungals, including fluconazole, voriconazole, itraconazole, amphotericin B, and caspofungin, with the highest resistance to fluconazole and the lowest resistance to caspofungin. The increasing prevalence of *Candida kefyr* in opportunistic infections, particularly among the immunocompromised, underscores the urgency for new treatment strategies and diligent antimicrobial stewardship. Our findings advocate for routine drug susceptibility testing to guide the selection of effective antifungal therapies and to monitor resistance trends, facilitating more effective management of candidiasis.

## Figures and Tables

**Table 1:** MIC ranges, MIC_90_, MIC_50_, and GM of MIC values (*μ*g/mL), for the 65 clinical isolates of *C. kefyr* included in the study.

Human	Minimum inhibitory concentration (MIC) (*μ*g/mL)														MIC & GM		
	Antifungals	0.03	0.06	0.125	0.25	0.5	1	2	4	8	16	32	64	Range	MIC_50_	MIC_90_	GM
*Healthy individuals (41)*																	
	CAS	0	1	6	4	13	10	5	1	0	1	—	—	0.06–16	0.5	2.0	0.5821
	FLU	0	0	2	1	6	3	8	2	5	7	0	7	0.125–≥64	4	64	4.0681
	ITR	1	1	5	14	7	0	0	1	0	12	—	—	0.03–≥16	0.25	16	0.9388
	VOR	5	6	8	2	4	3	1	1	1	10	—	—	0.03–≥16	0.25	16	0.5433
	AMB	0	0	0	1	14	22	4	0	0	0	—	—	0.25–2	1	1	0.8163

*Hematologic malignancy (9)*																	
	CAS	0	0	1	2	1	5	0	0	0	0	—	—	0.125–1	N/A	N/A	N/A
	FLU	0	0	0	1	6	1	0	1	0	0	0	0	0.25–4	N/A	N/A	N/A
	ITR	0	1	6	2	0	0	0	0	0	0	—	—	0.06–0.25	N/A	N/A	N/A
	VOR	7	2	0	0	0	0	0	0	0	0	—	—	0.03–0.06	N/A	N/A	N/A
	AMB	0	0	0	3	0	5	1	0	0	0	—	—	0.25–2	N/A	N/A	N/A

*Diabetes (8)*																	
	CAS	0	1	1	1	2	3	0	0	0	0	—	—	0.06–1	N/A	N/A	N/A
	FLU	0	0	0	3	3	1	0	0	1	0	0	0	0.25–8	N/A	N/A	N/A
	ITR	0	0	6	1	0	0	0	0	0	1	—	—	0.125–≥16	N/A	N/A	N/A
	VOR	5	2	0	0	0	0	0	1	0	0	—	—	0.03–4	N/A	N/A	N/A
	AMB	0	0	0	1	0	5	2	0	0	0	—	—	0.25–2	N/A	N/A	N/A

*HIV/AIDS (7)*																	
	CAS	0	0	1	1	3	2	0	0	0	0	—	—	0.125–1	N/A	N/A	N/A
	FLU	0	0	0	0	0	1	0	3	2	0	1	0	1–32	N/A	N/A	N/A
	ITR	0	0	0	4	2	0	0	1	0	0	—	—	0.25–4	N/A	N/A	N/A
	VOR	2	1	2	0	1	0	0	1	0	0	—	—	0.03–4	N/A	N/A	N/A
	AMB	0	0	0	0	3	3	1	0	0	0	—	—	0.5–2	N/A	N/A	N/A

*Note*. MIC_90_: lowest concentration at which 90% of the isolates are inhibited, MIC_50_: lowest concentration at which 50% of the isolates are inhibited, GM: geometric means. NA: not available.

**Table 2:** MIC ranges, MIC_50_, MIC_90_, and GM of MIC values (*μ*g/mL), for the 69 dairy isolates of *C. kefyr* included in the study.

Dairy product	Antifungals	Minimum inhibitory concentration (MIC) (*μ*g/mL)												MIC & GM			
		0.03	0.06	0.125	0.25	0.5	1	2	4	8	16	32	64	Range	MIC_50_	MIC_90_	GM
*Yogurt (49)*																	
	CAS	4	4	10	9	7	13	2	0	0	0	—	—	0.03–2	0.25	1.0	0.2861
	FLU	0	2	6	13	11	6	1	1	4	4	0	1	0.125–64	0.5	8.0	0.6917
	ITR	0	1	20	12	7	2	1	0	0	6	—	—	0.06–16	0.25	16	0.3745
	VOR	12	11	10	6	2	3	1	0	0	4	—	—	0.03–16	0.125	1.2	0.1537
	AMB	0	0	0	0	5	29	15	0	0	0	—	—	0.5–2	1.0	2.0	1.1521

*Milk (19)*																	
	CAS	0	2	4	3	2	8	0	0	0	0	—	—	0.06–1	0.5	1.0	0.3585
	FLU	0	0	2	10	1	2	0	0	4	0	0	0	0.125–8	0.25	8.0	0.5785
	ITR	1	1	6	8	3	0	0	0	0	0	—	—	0.03–0.5	0.25	0.5	0.1859
	VOR	8	6	1	1	2	1	0	0	0	0	—	—	0.03–1	0.06	0.5	0.0727
	AMB	0	0	0	1	1	12	5	0	0	0	—	—	0.25–2	1	2	1.0756

*Cheese (1)*																	
	CAS	0	0	0	0	0	1	0	0	0	0	—	—	N/A	N/A	N/A	N/A
	FLU	0	0	0	0	1	0	0	0	0	0	0	0	N/A	N/A	N/A	N/A
	ITR	0	0	0	1	0	0	0	0	0	0	—	—	N/A	N/A	N/A	N/A
	VOR	1	0	0	0	0	0	0	0	0	0	—	—	N/A	N/A	N/A	N/A
	AMB	0	0	0	0	0	1	0	0	0	0	—	—	N/A	N/A	N/A	N/A

*Note*. MIC_90_: lowest concentration at which 90% of the isolates are inhibited, MIC_50_: lowest concentration at which 50% of the isolates are inhibited, GM: geometric means. NA: not available.

**Table 3:** Interpretation of the MIC values (*μ*g/mL) based on ECV.

Antifungal drugs	Interpretation (ECV: *μ*g/mL)	Patients (24)			Healthy individuals (41)	Dairy products (69)	WT (%)
		HIV	Hematologic malignancy	Diabetes			
CAS	WT	2	3	3	11	36	41.4
	Non-WT	5	6	5	30	33	

FLU	WT	1	8	7	12	54	61.9
	Non-WT	6	1	1	29	15	

VOR	WT	2	7	5	7	21	31.3
	Non-WT	5	2	3	34	48	

ITR	WT	0	7	6	7	30	37.3
	Non-WT	7	2	2	34	39	

AMB	WT	6	8	6	37	49	79.1
	Non-WT	1	1	2	4	20	

**Table 4:** The reported ECVs for each drug against *C. kefyr* species.

Organism	Antifungal agents	ECVs (*μ*g/mL)	
		WT	Non-WT
*C. kefyr*	FLU	≤1	>1
	VOR	≤0.032	>0.032
	CAS	≤0.25	>0.25
	AMB	≤1	>1
	ITR	≤0.125	>0.125

## Data Availability

The data used to support the findings of this study are supplied by Shiraz University of Medical Sciences. Requests for access to these data should be made to Kamiar Zomorodian, zomorodian@sums.ac.ir or kzomorodian@gmail.com.
